# The relation between the storage symptoms before and after transurethral resection of the prostate, analysis of the risk factors and the prevention of the symptoms with solifenacin

**DOI:** 10.1590/S1677-5538.IBJU.2019.0227

**Published:** 2020-12-30

**Authors:** Timucin Sipal, Hakan Akdere

**Affiliations:** 1 Department of Urology Cerkezkoy State Hospital Tekirdag Turkey Department of Urology, Cerkezkoy State Hospital, Tekirdag, Turkey;; 2 Department of Urology Trakya University Medical Faculty Edirne Turkey Department of Urology, Trakya University Medical Faculty, Edirne, Turkey

**Keywords:** Transurethral Resection of Prostate, Risk Factors, Solifenacin Succinate

## Abstract

**Objective and Hypothesis:**

We aimed to investigate the reasons of storage symptoms ( SS) after transurethral resection of the prostate (TURP). The hypothesis was that a positive correlation would be identified between preoperative and postoperative SS in patients with undergoing TURP and starting early solifenacin treatment in patients with high preoperative SS would be reasonable. In addition, we aimed to analyze multiple other risk factors for post-TURP SS.

**Materials and Methods:**

A total of 160 patients undergoing TURP were prospectively evaluated and divided into two groups according to their OABS. Those with a score of ≥10 points were Group 1 (G1), and those with <10 points Group 2 (G2). In addition, patients in each group were randomly further divided into two subgroups: those who were started on 5 mg solifenacin succinate in the early postoperative period (G1/G2 A) and those who were not (G1/G2 B). In additions to SS Preop, perop and at the 3rd-month of postoperatively 14 variable were evaluated. The effects of these factors, surgery and the efficacy of an early medical treatment on the postoperative SS were investigated. LUTS were assessed by International Prostate Symptom Score (IPSS) and SS were assessed by sum of IPSS 2, 4 and 7 questionnaires (Storage, S- IPSS).

**Results:**

Preoperative IPSS and S-IPSS were significantly higher in G1 (p<0.001); there was a significant improvement at IPSS, S-IPSS, QoL score, Qmax, and PVR for all groups after surgery. Only preoperative S-IPSS was found to have significant effect on postoperative SS (p<0.001). There was a significant difference between G1A and G1B but no significant difference between G2A and G2B in terms of SS at postoperatively. In addition to this, prostatic volume was found smaller than non-symptomatic patients in de novo SS patients.

**Conclusion:**

TURP provides significant improvement in both storage and voiding symptoms. The predictive value of the preoperative S-IPSS on postop SS is significant. These results suggest that 5 mg solifenacin succinate treatment in the early postoperative period may be beneficial for patients with high preoperative SS and may not be beneficial in others. Small prostatic volume may bode ill for postoperative SS in the patients with de novo SS.

## INTRODUCTION

Transurethral resection of the prostate (TURP) is the most effective surgical treatment option for benign prostatic hyperplasia (BPH) and is still the gold standard and it has been shown to provide significant, sustained decrease in lower urinary tract symptoms (LUTS) and improvements in urodynamic parameters ( 1 ). However, voiding and storage symptoms (SS) during the postoperative period negatively affect quality of life ( 2 ). It has been reported that overactive bladder symptoms (OABS) persist in 20-35% of cases after TURP ( 3 ).

It is important for the surgeon and patients to know which group of patients is under risk for development of OABS after TURP. Several studies have shown that the success rates in the postoperative period were lower in patients with preoperative urodynamic detrusor overactivity (DO) and preoperative severe SS, although the data on this subject are contradictory ( 4 - 11 ). So there is no consensus in this issue, which therefore needs further studies.

Therefore, we conducted this prospective randomized study. We aimed to investigate the reasons of SS after transurethral resection of the prostate (TURP). The hypothesis was that a positive correlation would be identified between preoperative and postoperative SS in TURP and starting early solifenacin treatment in patients with severe preoperative SS would be reasonable. In addition, we aimed to analyze multiple other risk factors such as age, PSA, prostatic volume, energy sources, resection time, duration of postoperative catheterization, pathology results etc. for post-TURP SS including de-novo SS and nocturia.

## MATERIALS AND METHODS

### Patients

A total of 204 patients presented to our hospital between January 2014 and March 2017 who were candidates for TURP were enrolled. Following the approval of the study required local Ethics Committee (Issue: 42232755-799-E.54), patients were informed about the study and written consent forms were obtained.

Patients with moderate to severe symptom scores were included in the study. A total of 44 patients were excluded from the final analyses because they had a history of urologic surgery, prostate or bladder cancer pathology, bladder stone, suspected neurogenic disease, urinary retention, or ongoing anticholinergic medication preoperatively, and patients with urinary infection (n=5), urethral stricture formation (n=7), positive pathology results for cancer (n=3), clot retention on postoperative period (n=3), those who could not tolerate medical treatment (n=2) during the postoperative period and the those who missed the follow-up visits (n=6). After exclusion, the analysis was completed with remaining 160 patients.

**Figure 1:**
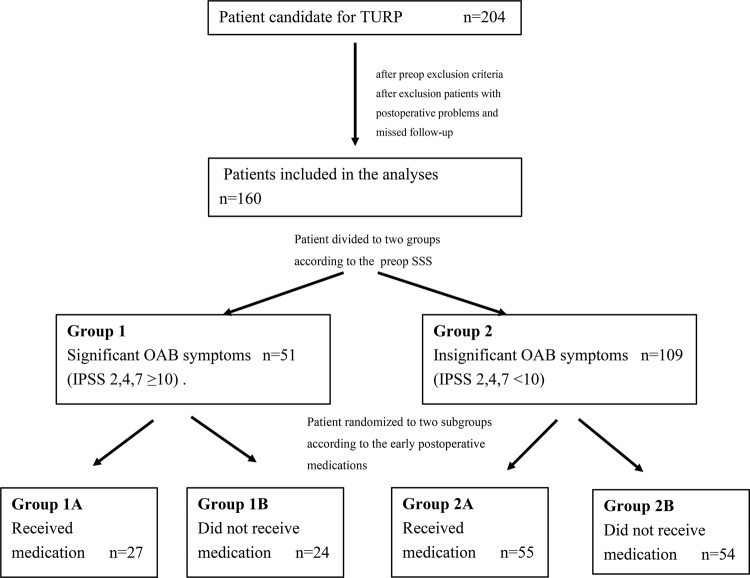
Study flow scheme A total of 204 patients candidate to TURP were enrolled the study. After exclusion, 160 patients completed 3 months follow-up study and the results were analyzed. Patients were divided two groups according to the preoperative SS severity. Those with a S-İPSS score of ≥10 points were included in Group 1 (G1), and those with <10 points in Group 2 (G2). In addition, patients in each group were randomly further divided into two subgroups: those who were started on 5mg solifenacin succinate in the early postoperative period (G1/G2 A) and those who were not (G1/G2 B). **TURP** = Transurethral prostatectomy; **IPSS** = İnternational prostate symptom scores; **SS** = storage symptoms.

Patient’s LUTS were assessed by International Prostate Symptom Score (IPSS) and SS scores were assessed by total scores of İPSS 2, 4 and 7 questionnaires (S-IPSS) because IPSS was validated in many languages around the World, safely used in previous studies, and its use is more convenient. Patients were divided into two groups according to their preoperative S-IPSS scores: those with significant SS scores (S-IPSS >10) in the preoperative period (G1) and those not (G2). Before the operation, patients in each group were randomly assigned to two subgroups according to their received medication: those who received medication in the postoperative period (G1A or G2A) and those did not (G1B or G2B). Solifenacin treatment was started after operation before discharge from the hospital. The workflow diagram is shown in Figure-1 .

Patients with a score of <8 points in the postoperative assessment or a reduction of more than 50% in S-IPSS (compared to their preoperative score) were considered as improvement.

In addition to S-IPSS, 17 other variables were analyzed ( Table-1 ). Surgery was performed using a 26Fr monopolar resectoscope (Karl Storz, Germany) and a 26Fr plasma kinetic bipolar resectoscope (Gyrus Agmi, US). The starting and finishing times of the resection were recorded. The choice of energy source was determined randomly at the intraoperative period. Solifenacin 5mg once daily was prescribed as postoperative medications in the study group before discharge from the hospital.

**Table 1:** Preoperative and perioperative data for the patient groups.

Characteristic	All	Group 1A	Group 1B	Group 2A	Group 2B	p
Age (year)	65.4 ± 7.8	67.3 ± 8.9	63.9 ± 5.3	65 ± 8.5	65.7 ± 7.6	0.328*
PSA ( ng/mL)	2.59 ± 3	3.7 ± 5	2.8 ± 3.6	2.1 ± 1.4	2.4 ± 2.5	0.548*
Prostate Size† (gr)	54.1 ± 12	50.8 ± 17.8	47.2 ± 15.6	57.6 ± 16.9	55.2 ± 14.1	0.047*
**Preoperative Parameters**						
IPSS	24.8 ± 5.8	28.7 ± 6.3	27.2 ± 6.5	23 ± 4.9	23.8 ± 5.2	**<0.001**
S-İPSS	7.4 ± 3.8	11.3 ± 1.9	12.5 ± 1.7	5.4 ± 2.6	5.2 ± 2.4	**<0.001**
QoL	5.19 ± 0.6	5.3 ± 0.6	5.2 ± 0.7	5.2 ± 0.7	5.1 ± 0.6	0.502
Qmax (mL/sn)	7 ± 3.3	7.9 ± 3.4	8.1 ± 3.2	6.4 ± 3.2	6.8 ± 3.4	0.097
PVR (mL)	61.8 ± 24	47.9 ± 15.7	49.04 ± 9.3	68.2 ± 23.3	68.2 ± 29.4	**<0.001**
Resection Time, min	45.5 ± 12.3	44.5 ± 15	44.3 ± 14.1	46.4 ± 10.6	45.8 ± 12.1	0.263*
Postop Cat ( day)	3.8 ± 0.9	3.9 ± 0.9	3.8 ± 0.9	3.9 ± 0.9	3.7 ± 0.9	0.491*
**Pathology Result, n (%)**						
BPH		15 (55.6)	12 (50.0)	26 (47.3)	24 (44.4)	0.823**
BPH, Prostatitis		12 (44.4)	12 (50.0)	29 (52.7)	30 (55.6)	
**Energy Source, n (%)**						
Bipolar		11 (40.7)	9 (37.5)	20 (36.4)	20 (37)	0.990**
Monopolar		16 (59.3)	15 (62.5)	35 (63.6)	34 (63)	

**post hoc paired comparison**	**Group 1 a vs. 1 b**	**Group 1 a vs. 2 a**	**Group 1 a vs. 2 b**	**Group 1 b vs. 2 a**	**Group 1 b vs. 2 b**	**Group 2 a vs. 2 b**
**Prostate size**	0.521	0.088	0.184	0.013	0.034	0.645 ***

Continuous variables are expressed as mean ± SD

* = Kruskal-Wallis test; ** = Fisher’s Exact test; *** = Mann-Whitney U p

**PSA** = Prostate specific antigen; I **PSS** = International prostate symptom score; **QoL** = Quality of life, **Qmax:** Maximum flow rate; **PMR** = Postvoid residual urine; Postop Cat = Duration of postoperative catheterization; **BPH** = Benign prostate hyperplasia; **SD** = standard deviation.

† = Patients in Group 1B has significantly lower prostate size compared to Groups 2A and 2B (p=0.013 and p=0.034, respectively).

The questionnaires were re-implemented at postoperative 3rd-month visit, pathology results were evaluated, and uroflowmetry and PVR were assessed.

### Statistical analysis

Mann-Whitney U test was used to compare two independent variables not fitting to normal distribution. The Wilcoxon test was used to compare two dependent variables not fitting to normal distribution between two groups. The Kruskal-Wallis test was used to compare the independent continuous variables not fitting to normal distributions between more than two groups. The χ2 test (or the Fisher Exact test where appropriate) was used to compare categorical variables between study groups. Statistical significance level was accepted as p<0.05. Analyses were performed using MedCalc Statistical Software version 12.7.7 (MedCalc Software BVBA, Ostend, Belgium; http://www.medcalc.org; 2013).

## RESULTS

### Preoperative and perioperative findings

Data for the preoperative and perioperative period are presented in Table-1 . No significant difference was found between groups in terms of age and preoperative PSA. The mean prostate volume was 50.5±15.1g in the study population. There was a significant difference between groups in terms of prostate volume (p=0.047) in multi-group comparison. Patients in Group 1B has significantly lower prostate size compared to Groups 2A and 2B (p=0.013 and p=0.034, respectively). However, no significant difference was found in paired group comparisons between Groups 1B-2A and 1B-2B (p=0.013 and p=0.034, respectively (post hoc paired comparison). No significant difference was found between the groups in terms of perioperative resection time, energy source, prostate tissue pathology results and postoperative catheterization times (Kruskal-Wallis test, Fisher’s Exact test, Mann-Whitney U test p >0.008, with Bonferroni correction). No difference in Qmax and QoL scores values were found between the groups in the preoperative period. The mean preoperative PVR was 61.8±24.7mL. Preoperative PVR in Group 2 was found significantly higher than Group 1 (p<0.001). The mean preoperative IPSS and S-IPSS scores were 26.1±4.7 and 7.4±5.8 in all patients, respectively. These were found significantly higher in Group 1 than Group 2 (p <0.001).

### Postoperative results

One hundred and sixty patients after the exclusion criteria were analyzed. Data for the postoperative period are shown in Table-2 . In all patients, the mean IPSS and S-IPSS scores were 8.14±6.9 points and 5.1±3.8 points at postoperative 3rd month, respectively. In all groups, significant improvements were found in the IPSS scores from the preoperative period to postoperative 3rd month (p <0.001). In the comparison of preoperative and postoperative S-IPSS scores, which represent the irritative symptoms, a significant improvement was found between Group 1A, 1B, and 2A (p<0.008) but not in Group 2B (p=0.126). The mean Qmax at postoperative 3rd month was 22.5±6.9. In all groups, significant improvements were found in Qmax (p<0.001). Postoperative QoL score was 2±1.2 in all patients. In all groups, significant improvements were found in QoL (p<0.001). The mean PVR at postoperative 3rd month was 22.4±16.4mL in all patients. Postoperative PVR was found significantly higher in Group 2 compared with Group 1. In the analysis of the effect of medical treatment on postoperative PVR, no difference was found between Groups A and B in post hoc paired comparison.

**Table 2:** Changes in the values of parameters from preoperative to postoperative 3rd months.

Variable	All	Group 1A	Group 1B	Group 2A	Group 2B	p*	Post-hoc comparisons***		
								*Preop*	*Postop*
**IPSS Preop**	24.8 ± 5.8	28.7 ± 6.3	27.2 ± 6.5	23.05 ± 4.9	23.8 ± 5.2	**<0.001**	Group 1A vs Group1B	0.399	**0.005**
**Postop 3-m**	8.14 ± 6.9	6.5 ± 2.3	12.2 ± 9.3	8.4 ± 8.4	7 ± 4.7	0.064	Group 1A vs Group 2A	**<0.001**	0.847
p**	**<0.001**	**<0.001**	**<0.001**	**<0.001**	**<0.001**		Group 1A vs Group 2B	**<0.001**	0.972
							Group 1B vs Group 2A	**0.001**	0.038
							Group 1B vs Group 2B	**0.005**	0.018
							Group 2A vs Group 2B	0.179	0.995
**IPSS247 Preop**	8.14 ± 6.9	11.3 ± 1.9	12.5 ± 1.7	5.4 ± 2.6	5.2 ± 2.4	**<0.001**	Group 1A vs Group1B	0.022	**<0.001**
**Postop 3-m**	5.1 ± 3.8	5.1 ± 2	7.6 ± 2.6	4.6 ± 4.9	4.5 ± 3.6	**0.001**	Group 1A vs Group 2A	**<0.001**	0.159
p**	**<0.001**	**<0.001**	**<0.001**	**0.034**	0.126		Group 1A vs Group 2B	**<0.001**	0.242
							Group 1B vs Group 2A	**<0.001**	**0.003**
							Group 1B vs Group 2B	**<0.001**	**<0.001**
							Group 2A vs Group 2B	0.652	0.522
**QoL Preop**	5.19 ± 0.6	5.3 ± 0.6	5.2 ± 0.7	5.2 ± 0.7	5.1 ± 0.6	0.502			
**Postop 3-m**	2.0 ± 1.2	2.1 ± 1.3	2.5 ± 1.2	2.04 ± 1.5	2.1 ± 1.2	0.547			
p**	**<0.001**	**<0.001**	**<0.001**	**<0.001**	**<0.001**				
**Qmax Preop**	7 ± 3.3	7.9 ± 3.4	8.1 ± 3.2	6.4 ± 3.2	6.8 ± 3.4	0.097			
**Postop 3-m**	22.5 ± 6.9	21.9 ± 7.1	25.2 ± 6.5	23.9 ± 5.5	22.1 ± 6.8	0.072			
p**	**<0.001**	**<0.001**	**<0.001**	**<0.001**	**<0.001**				
**PVR Preop**	61.8 ± 24	47.9 ± 15.7	49.04 ± 9.3	68.2 ± 23.3	68.2 ± 29.4	**<0.001**	Group 1A vs Group1B	0.940	0.061
**Postop 3-m**	22.4 ± 16.4	23.8 ± 16.6	14.3 ± 13.2	26.7 ± 17.1	20.9 ± 15.9	**0.003**	Group 1A vs Group 2A	**<0.001**	0.096
p**	**<0.001**	**<0.001**	**<0.001**	**<0.001**	**<0.001**		Group 1A vs Group 2B	**0.001**	0.956
							Group 1B vs Group 2A	**<0.001**	**<0.001**
							Group 1B vs Group 2B	**0.001**	0.038
							Group 2A vs Group 2B	0.516	0.028

* = Kruskal-Wallis test; ** = Wilcoxon test ; *** = Mann-Whitney U test

**IPSS** : International prostate symptom score, **QoL** : Quality of life, Qmax: Maximum flow rate, **PVR** : Post-void residual urine.

### Overview of storage symptoms

The mean postoperative 3rd-month S-IPSS score in all patients was 5.1±3.8; the number of patients with a score of pre-operative ≥8 points decreased from 74 to 36 in the postoperative period, equivalent to a 48.6% improvement. The mean S-IPSS scores were 5.1±2 in Group 1A, 7.6±2.6 in Group 1B, 4.6±4.9 in Group 2A, and 4.5±3.6 in Group 2B. Group 1B was found to be significantly different than other groups in terms of the improvement in storage symptoms. The proportions of patients with a 50% or more reduction in symptom score were 74.1% in Group 1A, 29% in Group 1B (p <0.01), 49% in Group 2A, and 42% in Group 2B (p=0.52). The proportion of patients with postoperative S-IPSS scores of <8 points were 96.3% in Group 1A, 54.2% in Group 1B, 74.5% in Group 2A, and 81.5% in Group 2B, significant improvements were found in all groups except for Group 1B (p <0.001). In Group 2, in which patients had low rate of preoperative SS, the rate of those with symptom scores of ≥8 points was 22% (n=24) in the postoperative period. This rate represents de novo effect of TURP on the storage symptoms (SS).

### Analysis of the factors that may be effective on SS

The preoperative factors that may be effective on postoperative SS were analyzed by a multiple linear regression model, and the preoperative S-IPSS score was found to have significant effect (p<0.001). One-unit increase in the preoperative S-IPSS score increases postoperative S-IPSS score by 0.609 points. In addition to the regression analysis, there was a significant, moderate positive correlation (Spearman’s rho p<0.001) between the postoperative and preoperative S-IPSS scores. All other variables including age, PSA, prostate size, duration of resection, pathology results, energy source, and catheter duration were found to have no effect on the outcome (p>0.05 for all).

### The analyses of nocturia and de novo SS

The results of the nocturia analyses (IPSS-7) show that its scores significantly decreased after operations in all groups (p <0.001). There is a significant difference between G1 and G2 patients at the preoperative and postoperative period (p <0.001). Pairwise comparison of the groups shows that there is a significant difference between group 1A and group 1B at the postoperative period (p=0.024) whereas there is no difference between group 2A and 2B (p=0.251).

De novo OABS was defined by a rising of storage symptoms after operations. Patients with mild preoperative SS (S-IPSS is lower than 8 points) and severe postoperative SS (S-IPSS is higher than 10 points) were considered have de novo SS. In this respect, only the patients in group 2 were evaluated because these patients did not have significant SS preoperatively. The analyses showed that 22% of all patients have experienced de novo SS (35 of the 160). The comparisons between the subgroups showed that there were significant differences in terms of preoperative S-IPSS scores (<0.001), preoperative Q max values (mL/sec) (p<0.001) and prostatic volumes (mL) (p<0.003).

## DISCUSSION

Transurethral resection of the prostate ( TURP) is still the gold standard surgical treatment option for relief storage and voiding symptoms related to BPH and cause significant, sustained decrease in lower urinary tract symptoms (LUTS) including nocturia and improvements in urodynamic parameters ( 1 ).

Similarly, in our study the improvement of İPSS, S-IPSS, İPSS-7, QOL, PVR and Q max were statistically significant(p<0.001) All of these results are consistent with previous studies and demonstrate the effectiveness of traditional TURP in treating the symptoms of BPH.

Re-innervation of the bladder and restoration of detrusor stability as a result of the elimination of the obstruction and decreases of PVR were suggested as an effective factor to explain the reasons for the improvement in storage symptoms after TURP ( 12 , 13 ). But in contrast to voiding symptoms, the storage symptoms do not clearly correlate with BOO, and may also occur independently of BOO. For this reason, OAB symptoms may persist after pharmacological and surgical treatment of BOO ( 14 , 15 ).

In a study by De Nunzio et al., DO was shown to decrease from 68% to 31% within 2 years after prostatectomy (54% regression) ( 15 ). In our study, the rate of improvement in SS at postoperative 3rd month was found as 48.6%. In the separate patient groups, this rate was 74.1% (Group 1A), 29% (Group 1B), 49% (Group 2A), or 42% (Group 2B). Significant improvements were observed in the groups (p <0.001) other than Group 1B.

There are controversial results in the literature about the effect of preoperative SS and DO on the postoperative SS. Seki et al. have shown that DO was an independent predictor of postoperative restoration and that a severe preoperative SS score adversely affects the postoperative QoL score ( 4 ). Machino et al. reported that the cases with persistent DO had detrusor instability at a rate of 60% in the preoperative period ( 5 ). Similarly, Antunes et al. reported 66.7% persistence of preoperative DO complaints in the postoperative period ( 6 ).

There are also studies showing that SS scores other than urodynamic DO are also useful in predicting postoperative outcomes. In a retrospective study by Zhao et al. involving 128 BPH patients, the outcomes of patients with mild preoperative SS were shown to be much better than those with moderate and severe symptoms ( 7 ). In Choi et al.’s study with 116 patients, multivariate analysis has shown that poor initial SS were a risk factor for persistent SS in the postoperative period (OR=8.32) ( 10 ). In a study by Porru et al. including 60 patients, postoperative symptom scores were shown to get worse in patients with significant preoperative SS (p=0.001) ( 11 ).

Thus, we considered high SS scores as the preoperative risk factor in our patients rather than the presence of urodynamic DO and we did not perform urodynamic studies in the lack of absolute indications. Furthermore, it has been shown that SS strongly correlate with the urodynamic OD [10, 11, 13, and 21]. İn parallel with, postoperative storage complaints were found higher in patients with significant preoperative storage complaints in our study, (p <0.001). In contrast, some other studies have reported that preoperative DO does not predict postoperative SS ( 16 , 17 ).

When we address the issue of de novo SS, it is known that post-TURP SS continues in 20-25% or even appears de novo ( 3 ). Similarly, the rate of de novo SS was observed in 22% of our patients. Permanent changes due to BOO in the bladder were proposed among the possible mechanisms for de novo SS ( 18 ). Persistence of DO was found in the elderly cases, in a previous study and they explain this situation by the aging bladder that may lead to functional change and persistent DO symptoms ( 6 ). However, we did not observe an effect of age on persistent SS in our study (p=0.34).

In one study, a correlation has been found between prostatic size and postoperative storage symptom severity, suggesting that patients with small prostate (less than 30g) are under risk ( 19 ). Similarly in our study, there were significant differences in term of prostatic volumes (mL) (p <0.003), preoperative S-IPSS scores (<0.001), preoperative Qmax values (mL/sec) (p<0.001) but such a relationship was found only in patients with de novo SS, not others (p=0.9).

Because of small prostate it may be argued that the cause of underlying pathology is OAB rather than BOO in the group 1B to explain their LUTS. And the statistical differentiation in the prostatic volume may reflect the fact that the underlying pathology is not the prostate, but rather the problem. Without urodynamics (cystometrogram and pressure flow study), this is unclear. And it does not change the fact that it seems medical therapy helps improve postoperative LUTS. But, in addition to the improvement of storage symptoms, excellent improvement was seen in the voiding symptoms after TURP (P <0.001). And the mean prostatic volume and PVR (47.2±15.6gr 49.04±9.3cc, respectively) were greater than normal reference values in group 1 and there is not difference between the two groups in term of Qmax (p=0.097). These suggested that underlying pathology may not only be OAB but also BOO.

When we compare the groups that received medical treatment, the rate of patients with de novo SS was different between G2A and G2B 30% and 33.3%, respectively, but not statistically significant (p=0.8). It means that the effect of solifenacin on de novo SS in this group is not clear, which might be explained by the small sample size of the subgroups.

Nocturia has been recognized as one of the most bothersome symptoms of LUTS/BPO and adversely affects the quality of life. Previous studies have convincingly shown that TURP has beneficial effect on nocturia ( 4 , 13 ). In the current study, nocturia symptoms were significantly decreased in all groups after the operation (p <0.001). The pairwise comparison of the groups showed that there was significant difference between group 1A and group 1B in the postoperative period (p=0.024) whereas there was no difference between groups 2A and 2B (p=0.251). These results led us to think that the patients with high preoperative nocturia and S-IPSS scores may be under risk for postoperative nocturia symptoms and that early medical treatment adds extra benefit in high-risk patients.

When we investigate other intra-operatorative factors for post TURP SS, bipolar TURP has previously been shown to reduce postoperative SS ( 20 ). In our study, however, there was no significant difference between the two energy sources (p=0.6). In one study examining the effect of the pathology involved on SS, Nunzio et al. found a 55% reduction after TURP in the storage symptoms of patients with chronic prostatitis pathology ( 21 ). However, no such difference was found in our study (p=0.6).

Antimuscarinics are the first-line treatment for SS in men ( 22 ). We used 5mg solifenacin succinate as a prophylactic treatment on postoperative SS in different groups, which have been shown to be effective in OAB treatment. The improvement in postoperative SS of patients in Group 1A who used solifenacin, was significantly higher than in Group 1B (medication-free group) (p<0.001). However, solifenacin use did not result in significant improvement in postoperative SS in patients of Group 2 whose preoperative SS were not obvious (p <0.522). This result suggests that SS may be frequent in patients with preoperative SS, and that early initiation of anticholinergic treatment is beneficial only in this group of patients, but not for other patients.

Only a few studies have been found investigating the benefit of early medical treatment for the prevention of postoperative SS. Iselin et al. have shown that early oxybutynin treatment after TURP improved SS in the first week except for nocturia ( 23 ). In a randomized study, Tehranci et al. found that postoperative treatment with tolterodine 2mg twice daily had significantly improved SS after TURP compared with placebo and reduced the need for analgesics (p=0.001 and p=0.036, respectively) without a significant difference in side effects ( 24 ). Shin et al. compared the patients who underwent TURP with no postoperative medical treatment (Group 1), with postoperative tamsulosin 0.2mg per day (Group 2), and postoperative solifenacin 5mg+tamsulosin 0.2mg per day (Group 3). They found that SS were lower in Group 3 compared to Group 2, but the improvements in storage and voiding symptoms and QoL in Groups 2 and 3 were not significantly different than Group 1 ( 25 ).

In all three studies above-mentioned, randomized treatment was initiated to eliminate that may occur after TURP. However, the initiation of medical treatment not randomly but in patients who most likely will develop these symptoms would be more rational both in terms of efficacy, cost effectiveness and safety. From this point of view, our study is the first to examine the benefit of the early postoperative treatment started only in the patients with severe SS development risk. In our study, postoperative SS in Group 1A, which received anticholinergic treatment, were found to be significantly lower than in Group 1B, which did not receive treatment. However, no such difference was observed between Group 2A (with treatment) and Group 2B (without treatment), both of which did not have significant SS in the preoperative period. This clearly demonstrates that it would be more beneficial to give early medical treatment only to the patient group with preoperative SS. However, no significant difference was found between groups A and B in terms of treatment-related improvements in the PVR.

The limitations of this study were as follows: the study was conducted at a single center, urodynamic studies were not performed in preoperative and postoperative periods routinely, small number of patients, and relatively short follow-up time. Finally, we did not use a specific questionnaire focusing on urgency symptoms such as the OABSS (Overactive Bladder Symptom Score).

## CONCLUSIONS

TURP provides significant improvement in both storage and voiding symptoms. Severe preoperative S-İPSS scores have a predictive value for the storage complaints after TURP. Small prostate may predict postoperative SS and reflect the underlying OAB related pathology in the patients with de novo SS. With this in mind, 5mg solifenacin succinate treatment started early in the postoperative period seems to be beneficial only in patients with significant preoperative storage complaints but not in others.

## References

[1] 1. Thomas AW, Cannon A, Bartlett E, Ellis-Jones J, Abrams P. The natural history of lower urinary tract dysfunction in men: minimum 10-year urodynamic followup of transurethral resection of prostate for bladder outlet obstruction. J Urol. 2005;174:1887-91. 10.1097/01.ju.0000176740.76061.24 16217330

[2] 2. Rassweiler J, Teber D, Kuntz R, Hofmann R. Complications of transurethral resection of the prostate (TURP)--incidence, management, and prevention. Eur Urol. 2006;50:969-79; discussion 980. 10.1016/j.eururo.2005.12.042 16469429

[3] 3. Taylor J, Harrison SC, Assassa RP, McGrother CW; Leicestershire MRC Incontinence Study Group. The pattern and progression of lower urinary tract symptoms after transurethral prostatectomy compared with those seen in the general population. Eur Urol. 2007;51:1023-9; discussion 1029-30. 10.1016/j.eururo.2006.10.012 17081677

[4] 4. Seki N, Takei M, Yamaguchi A, Naito S. Analysis of prognostic factors regarding the outcome after a transurethral resection for symptomatic benign prostatic enlargement. Neurourol Urodyn. 2006;25:428-32. 10.1002/nau.20262 16721841

[5] 5. Machino R, Kakizaki H, Ameda K, Shibata T, Tanaka H, Matsuura S, et al. Detrusor instability with equivocal obstruction: A predictor of unfavorable symptomatic outcomes after transurethral prostatectomy. Neurourol Urodyn. 2002;21:444-9. 10.1002/nau.10057 12232878

[6] 6. Antunes AA, Iscaife A, Reis ST, Albertini A, Nunes MA, Lucon AM, et al. Can we predict which patients will experience resolution of detrusor overactivity after transurethral resection of the prostate? J Urol. 2015;193:2028-32. 10.1016/j.juro.2014.12.095 25583645

[7] 7. Zhao YR, Liu WZ, Guralnick M, Niu WJ, Wang Y, Sun G, et al. Predictors of short-term overactive bladder symptom improvement after transurethral resection of prostate in men with benign prostatic obstruction. Int J Urol. 2014;21:1035-40. 10.1111/iju.12482 24825248

[8] 8. Kageyama S, Watanabe T, Kurita Y, Ushiyama T, Suzuki K, Fujita K. Can persisting detrusor hyperreflexia be predicted after transurethral prostatectomy for benign prostatic hypertrophy? Neurourol Urodyn. 2000;19:233-40. 10.1002/(sici)1520-6777(2000)19:3<233::aid-nau4>3.0.co;2-m 10797580

[9] 9. Madersbacher S, Marberger M. Is transurethral resection of the prostate still justified? BJU Int. 1999;83:227-37. 10.1046/j.1464-410x.1999.00908.x 10233485

[10] 10. Choi H, Kim JH, Shim JS, Park JY, Kang SH, Moon du G, et al. Prediction of persistent storage symptoms after transurethral resection of the prostate in patients with benign prostatic enlargement. Urol Int. 2014;93:425-30. 10.1159/000357626 25300422

[11] 11. Porru D, Jallous H, Cavalli V, Sallusto F, Rovereto B. Prognostic value of a combination of IPSS, flow rate and residual urine volume compared to pressure-flow studies in the preoperative evaluation of symptomatic BPH. Eur Urol. 2002;41:246-9. 10.1016/s0302-2838(02)00021-0 12180223

[12] 12. Cumming JA, Chisholm GD. Changes in detrusor innervation with relief of outflow tract obstruction. Br J Urol. 1992;69:7-11. 10.1111/j.1464-410x.1992.tb15448.x 1737256

[13] 13. Margel D, Lifshitz D, Brown N, Lask D, Livne PM, Tal R. Predictors of nocturia quality of life before and shortly after prostatectomy. Urology. 2007;70:493-7. 10.1016/j.urology.2007.05.001 17905104

[14] 14. Athanasopoulos A, Chapple C, Fowler C, Gratzke C, Kaplan S, Stief C, et al. The role of antimuscarinics in the management of men with symptoms of overactive bladder associated with concomitant bladder outlet obstruction: an update. Eur Urol. 2011;60:94-105. 10.1016/j.eururo.2011.03.054 21497434

[15] 15. de Nunzio C, Franco G, Rocchegiani A, Iori F, Leonardo C, Laurenti C. The evolution of detrusor overactivity after watchful waiting, medical therapy and surgery in patients with bladder outlet obstruction. J Urol. 2003;169:535-9. 10.1097/01.ju.0000045600.69261.73 12544303

[16] 16. Nitti VW, Kim Y, Combs AJ. Voiding dysfunction following transurethral resection of the prostate: symptoms and urodynamic findings. J Urol. 1997;157:600-3. 8996367

[17] 17. Jensen KM, Jørgensen TB, Mogensen P. Long-term predictive role of urodynamics: an 8-year follow-up of prostatic surgery for lower urinary tract symptoms. Br J Urol. 1996;78:213-8. 10.1046/j.1464-410x.1996.11012.x 8813916

[18] 18. Mitterberger M, Pallwein L, Gradl J, Frauscher F, Neuwirt H, Leunhartsberger N, et al. Persistent detrusor overactivity after transurethral resection of the prostate is associated with reduced perfusion of the urinary bladder. BJU Int. 2007;99:831-5. 10.1111/j.1464-410X.2006.06735.x 17244278

[19] 19. Kang YJ, Kim KH, Seo Y, Lee KS. Effect of Transurethral Resection of the Prostate on Storage Symptoms in Patients with Benign Prostatic Hyperplasia of Less than 30 mL. World J Mens Health. 2013;31:64-9. 10.5534/wjmh.2013.31.1.64 PMC3640155 23658868

[20] 20. Issa MM. Technological advances in transurethral resection of the prostate: bipolar versus monopolar TURP. J Endourol. 2008;22:1587-95. 10.1089/end.2008.0192 18721041

[21] 21. De Nunzio C, Brassetti A, Gacci M, Finazzi Agrò E, Carini M, Presicce F, et al. Patients With Prostatic Inflammation Undergoing Transurethral Prostatic Resection Have a Larger Early Improvement of Storage Symptoms. Urology. 2015;86:359-65. 10.1016/j.urology.2015.04.048 26194294

[22] 22. Chapple CR, Rechberger T, Al-Shukri S, Meffan P, Everaert K, Huang M, et al. Randomized, double-blind placebo- and tolterodine-controlled trial of the once-daily antimuscarinic agent solifenacin in patients with symptomatic overactive bladder. BJU Int. 2004;93:303-10. 10.1111/j.1464-410x.2004.04606.x 14764127

[23] 23. Iselin CE, Schmidlin F, Borst F, Rohner S, Graber P. Oxybutynin in the treatment of early detrusor instability after transurethral resection of the prostate. Br J Urol. 1997;79:915-9. 10.1046/j.1464-410x.1997.00138.x 9202559

[24] 24. Tehranchi A, Rezaei Y, Shojaee R. Tolterodine to relieve urinary symptoms following transurethral resection of the prostate: a double-blind placebo-controlled randomized clinical trial. Korean J Urol. 2014;55:260-4. 10.4111/kju.2014.55.4.260 PMC3988437 24741415

[25] 25. Shin YS, Zhang LT, You JH, Choi IS, Zhao C, Park JK. Efficacy and safety of tamsulosin hydrochloride 0.2 mg and combination of tamsulosin hydrochloride 0.2 mg plus solifenacin succinate 5 mg after transurethral resection of the prostate: a prospective, randomized controlled trial. Clin Interv Aging. 2016;11:1301-1307. 10.2147/CIA.S115042 PMC5034924 27698559

[26] 26. Hyman MJ, Groutz A, Blaivas JG. Detrusor instability in men: correlation of lower urinary tract symptoms with urodynamic findings. J Urol. 2001;166:550-2; discussion 553. 10.1016/s0022-5347(05)65982-4 11458066

[27] 27. Lewis AL, Young GJ, Abrams P, Blair PS, Chapple C, Glazener CMA, et al. Clinical and Patient-reported Outcome Measures in Men Referred for Consideration of Surgery to Treat Lower Urinary Tract Symptoms: Baseline Results and Diagnostic Findings of he Urodynamics for Prostate Surgery Trial; Randomised Evaluation of Assessment Methods (UPSTREAM). Eur Urol Focus. 2019;5:340-50. 10.1016/j.euf.2019.04.006 31047905

[28] 28. Andersson KE. LUTS treatment: future treatment options. Neurourol Urodyn. 2007;26(6 Suppl):934-47. 10.1002/nau.20500 17696154

